# Evolutionary proteomics identifies amino acids essential for ligand-binding of the cytokinin receptor CHASE domain

**DOI:** 10.1186/1471-2148-7-62

**Published:** 2007-04-17

**Authors:** Alexander Heyl, Klaas Wulfetange, Birgit Pils, Nicola Nielsen, Georgy A Romanov, Thomas Schmülling

**Affiliations:** 1 Institute of Biology/Applied Genetics, Free University of Berlin, Albrecht-Thaer-Weg 6, 14195 Berlin, Germany; 2 Department of Bioinformatics, Biocenter, Julius Maximilian University, 97074 Würzburg, Germany; 3 Institute of Plant Physiology, Russian Academy of Sciences, 127276 Moscow. Russia

## Abstract

**Background:**

In plants the hormone cytokinin is perceived by members of a small cytokinin receptor family, which are hybrid sensor histidine kinases. While the immediate downstream signaling pathway is well characterized, the domain of the receptor responsible for ligand binding and which residues are involved in this process has not been determined experimentally.

**Results:**

Using a live cell hormone-binding assay, we show that cytokinin is bound by a receptor domain predicted to be extracellular, the so called CHASE (cyclases, histidine kinase associated sensory extracellular) domain. The CHASE domain occurs not only in plant cytokinin receptors but also in numerous orphan receptors in lower eukaryotes and bacteria. Taking advantage of this fact, we used an evolutionary proteomics approach to identify amino acids important for cytokinin binding by looking for residues conserved in cytokinin receptors, but not in other receptors. By comparing differences in evolutionary rates, we predicted five amino acids within the plant CHASE domains to be crucial for cytokinin binding. Mutagenesis of the predicted sites and subsequent binding assays confirmed the relevance of four of the selected amino acids, showing the biological significance of site-specific evolutionary rate differences.

**Conclusion:**

This work demonstrates the use of a bioinformatic analysis to mine the huge set of genomic data from different taxa in order to generate a testable hypothesis. We verified the hypothesis experimentally and identified four amino acids which are to a different degree required for ligand-binding of a plant hormone receptor.

## Background

The plant hormone cytokinin is required for many fundamental processes and developmental programmes such as cell division, shoot branching, root development and senescence [1]. For the model plant *Arabidopsis thaliana *it has been shown that the cytokinin signal is perceived by members of the cytokinin receptor family, which are sensor histidine kinases [2-4]. Mutational analysis of the three cytokinin receptors (AHK2, AHK3, CRE1/AHK4) revealed that they act redundantly, but are absolutely required for normal cytokinin perception and plant growth [5-8]. In the current model, it is predicted that the hormone binds to the *Arabidopsis *histidine kinase receptors (AHKs) via an extracellular ligand binding domain, the so-called CHASE (cyclases/histidine kinases associated sensory extracellular) domain [9,10]. The CHASE domain, about 250 amino acids long, is exclusively found between two transmembrane regions as the N-terminal part of adenylyl cyclases, diguanylate cyclases or histidine kinases in a number of eukaryotes and numerous bacteria. It includes, for example, the spore differentiation factor, DhkA, and the osmosensing receptor-adenylyl cyclase ACG, which regulates spore dormancy, from the slime mold *Dictyostelium discoideum*. DhkA recognizes a small peptide, SDF-2 [11], and it was proposed that ACG binds discadenine [12]. Thus the CHASE domain is believed to bind diverse low molecular weight ligands. However, the ligand and its cognate receptor are only known in a few cases. Among higher eukaryotes the domain is found only in plants as part of specific sensor histidine kinases, the cytokinin receptors. It was proposed that plants acquired the CHASE domain through their chloroplasts, which have a cyanobacterial ancestry [9,10].

The binding of cytokinin to the receptor is thought to cause a conformational change leading to the autophosphorylation of a conserved histidine residue in the cytosolic part of the receptor. Subsequently, the signal is transferred to a canonical aspartate within the C-terminal part of the protein and transduced further by a multi-step two-component signaling system (for recent reviews see [13-15]).

The cytokinin binding activity of full-length CRE1/AHK4 was shown before by several different types of assays [3,4,16,17]. Although the CHASE domain is suspected of being the ligand binding domain, no systematic approach has been made with any cytokinin receptor to identify the binding domain unequivocally. Once a binding domain has been determined, the next step of the characterization is to identify functional amino acid residues. In this report we describe a novel knowledge-based approach that uses sequence information from distantly related organisms to predict putative functionally relevant sites in the ligand binding domain. The bioinformatics method was based on detecting differences in the evolution of individual amino acid sites between the CHASE domains of the different protein subclasses. The underlying premise was that a slower evolutionary rate of a given amino acid position, e.g. the conservation of a different amino acid in plants versus other organisms, would identify important positions for receptor function. These positions are putatively important in binding the plant-specific ligand, which is thought to be different from the other subgroups.

Thus the aims of this study were twofold: (i) mapping of the ligand binding domain of CRE1/AHK4 and (ii) identifying amino acids crucial for the binding of cytokinin to the receptor. Using a binding assay we provide direct experimental evidence that cytokinin is bound via the CHASE domain of CRE1/AHK4. The substitution of four of the five amino acids, which were predicted by evolutionary analysis to be important for a functional ligand-binding domain, caused a clear change in the ligand binding, in this case a complete loss of, or strongly reduced, cytokinin binding. This demonstrates the power of combining bioinformatic predictions with experimental validation, which have been proven to be a very useful tool in other subjects in the past [18,19]. Thus the data further underpin the general potential of evolutionary proteomics to identify functionally relevant sites in proteins of known or even of unknown function.

## Results

### CRE1/AHK4 binds cytokinin via the CHASE domain

**Figure 1:**
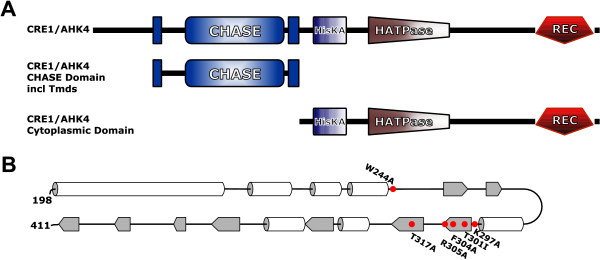
Domain structure of CRE1/AHK4 and secondary structure prediction of the CHASE domain. (A) The domain structure of the full-length protein and different truncated versions of CRE1/AHK4 used in this study. (B) The predicted secondary structure of the CHASE domain with the amino acid substitutions tested in the cytokinin binding assay marked in red. White tubes represent α-helices and grey areas β-sheets. Secondary structure prediction was done by PSIPred v2.4 [43]. Abbreviations: CHASE, cyclases, histidine kinase associated sensory extracellular; HisKA, histidine kinase A domain; HATPase, histidine kinase-like ATPase; Rec, receiver domain; Tmds, transmembrane domains.

**Figure 2:**
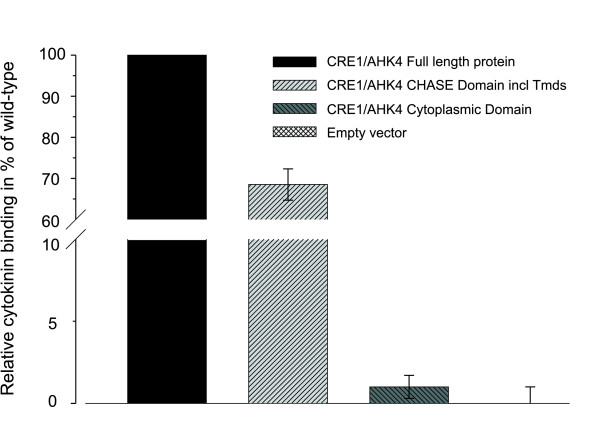
The CHASE domain of CRE1/AHK4 is necessary and sufficient for cytokinin binding. *In vitro *binding of *trans*-[2-3H]zeatin to full length CRE1/AHK4 protein or different domains of the receptor overexpressed in *E. coli *BL21. Bacterial cells were assayed for specific *trans*-[2-3H]zeatin binding. Data are mean ± s.d.; n = 4.

The cytokinin binding domain of CRE1/AHK4 was mapped by expressing the cytoplasmic part or the CHASE domain with the adjacent transmembrane domains as GST-fusion proteins in *E. coli *(Fig. 1A). The cytokinin binding capacity was tested in an *in vivo *binding assay [17] and compared to the binding capacity of the full-length protein in the same experimental setup. The full-length protein showed the highest relative *trans*-zeatin binding (Fig. 2). The binding capacity was slightly lower in the truncated version of CRE1/AHK4 consisting of the CHASE domain and the flanking transmembrane domains. The cytoplasmic domain and the empty vector control showed very weak or no binding (Fig. 2). The results of the binding assay indicate that the CHASE domain is the cytokinin binding domain of CRE1/AHK4.

### Evolutionary analysis of CHASE-subclasses reveals putative cytokinin-binding residues

**Figure 3:**
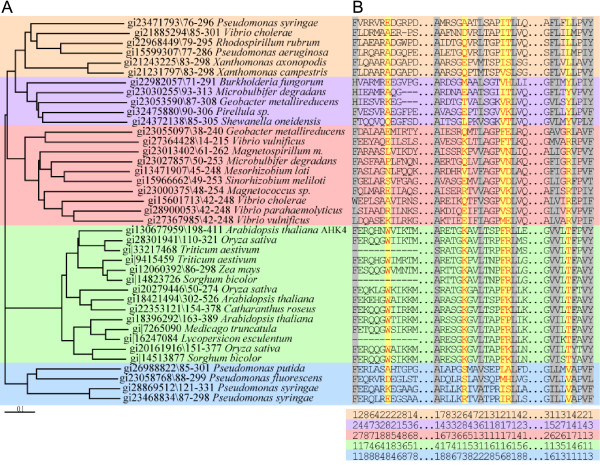
Phylogenetic tree and alignment of CHASE domains. (*A*) Phylogenetic tree of CHASE domains from five different subgroups (see Additional file 1 for all subgroups). Sequences of CHASE domains used for tree building are labelled with gi numbers and the start and end position of the respective CHASE domain. (*B*) Section of CHASE family alignment containing sequences used for the evolutionary rate analysis of the individual amino acids. The evolutionary site rate categories for each subgroup are given below the alignment, ranging from 1 (slow rate of evolution) to 8 (fast rate of evolution). Amino acid positions selected for experimental analysis (W244, K297, F304, R305, T317) are highlighted in orange, positions that are conserved throughout all CHASE sequences in grey. Interruptions of the alignment are indicated by two dots, gaps in the alignment by dashes. (See Additional file 1 for full sequence alignment). The selected blocks relate to the CRE1/AHK4 sequence 238–249, 292–308 and 313–321, respectively.

Sequences containing the CHASE domain were identified in plants, slime-molds, cyanobacteria and proteobacteria and a multiple sequence alignment was generated for the region spanning the domain (Figure 3B and Additional file 1; for details see Material and Methods). The phylogenetic tree reveals several distinct subgroups, one of which includes all plant sequences (Figure 3A). Our computational approach to identify functional important positions is based on the assumption that CHASE domains found in plants recognize a different class of ligands (cytokinin) than bacterial domains and that these functional differences can be detected by a change in evolutionary rates of amino acid substitutions. A candidate position should have a slow evolutionary rate in plants and a fast evolutionary rate in other subgroups or a slow evolutionary rate in all subgroups, but then the subgroups should be conserved in a different amino acid to distinguish the plant sequences from bacterial sequences. The slime-mold sequences were not included in this analysis because their subgroup contained only two sequences and was not supported by a high bootstrap value. Among the positions that were derived from the analysis of bacterial and plant sequences and fulfilled the above criteria we selected five positions, which show the strongest evidence for functional divergence and thus, are likely to be important in cytokinin binding: Position T317 of CRE1/AHK4 is not only slowly evolving among plant sequences but also among all investigated bacterial subgroups (evolutionary site rate category 1; Fig. 3B). However, in each subgroup, this site is occupied by a different amino acid with varying biochemical properties. Only in the plant subgroup, this position is occupied by an amino acid with a hydroxyl containing side chain (threonine). The evolutionary rate of CRE1/AHK4 positions W244 and K297 is very low in the plant CHASE sequences compared to that of the CHASE domains of the other subgroups, indicating that these positions might be important in plants. The amino acids at positions 304 and 305 have different biochemical properties and evolutionary rates in the different subgroups (Fig. 3B). Position 304 is occupied by phenylalanine only in the plant sequences while the bacterial subgroups have mostly aliphatic amino acids as a residue in this position. At position 305, the plant subgroup has either a basic amino acid or a proline, while in the other subgroups the class of amino acids is not conserved. It should be noted that position 301, a functional important residue of the CRE1/AHK4 CHASE domain, did not fulfil our criteria, because some bacterial sequences are conserved in the same or a similar amino acid. In fact, an allele mutated in this position, which leads to an amino acid change to isoleucine, is known as *wooden leg (wol) *and was discovered in a screen for altered root morphology [20]. Subsequent analysis of this only known mutation of the CHASE domain in plants revealed the complete loss of cytokinin binding of the mutant protein [4]. We included this mutation as a positive control in our analysis.

### Specific amino acid substitutions lead to significant changes in cytokinin binding

**Figure 4:**
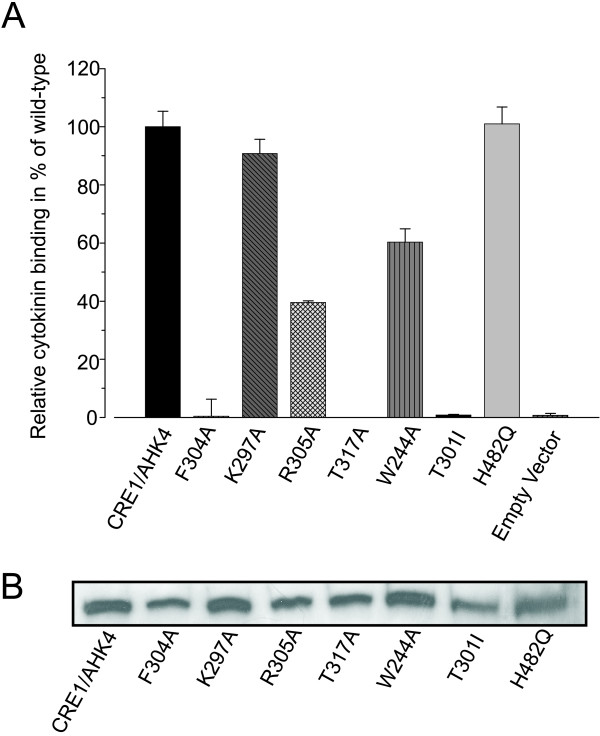
Identification of amino acid residues of the CRE1/AHK4 CHASE domain important for ligand binding. (*A*) Effect of point mutations in the CRE1/AHK4 CHASE domain on the specific binding of *trans*-[2-3H]zeatin. For the localization of the mutated sites see Fig. 1B. H482Q is a control carrying a mutated histidine residue of the cytoplasmic domain. Data are means ± S.D. from measurements with two different *E. coli *clones for each construct. (*B*) Western blot with whole protein extracts of an aliquot of *E. coli *cells used for the binding assay shown in (*A*). For protein detection a mouse-anti-GST antibody (GST B-14, Santa Cruz Biotechnology) was used.

The selected amino acids were substituted by alanine. Subsequently the ability of the mutated CHASE domains as part of the full length protein was tested for cytokinin binding activity. As controls, the wild-type CRE1/AHK4 protein showed strong cytokinin binding, while the empty vector did not show any cytokinin binding in the assay (Fig. 4A). We also confirmed that the known mutation (T301I) in the CHASE domain causes loss of cytokinin-binding activity [4] (Fig. 4A). In contrast and as expected, the substitution of the canonical histidine of the cytoplasmic histidine kinase domain by glutamine (H482Q), which abolishes the receptor's signaling capacity [21], does not alter the cytokinin binding capacity of the receptor (Fig. 4A).

Two of the candidate residues, namely F304A and T317A led to a complete abolishment of ligand binding (Fig. 4A). Two other amino acid substitutions – W244A and R305A – resulted in strongly reduced binding capacity, approximately 60% and 40% of the CRE1/AHK4 wild-type control, respectively (Fig. 4A). In contrast, the substitution of K297A caused only a slight decrease of the cytokinin binding of the mutant protein compared to the wild-type CRE1/AHK4. The protein level of all GST-fusion proteins was checked by Western blot and did not show significant differences (Fig. 4B).

## Discussion

### CRE1/AHK4 binds cytokinin via the CHASE domain

In this study we investigated how the plant hormone cytokinin is recognized by its receptor. It has been hypothesized that cytokinin is bound by the CHASE domain of the receptors [2-4,9,10], but no experiment has been performed to prove this hypothesis. Testing truncated versions of a protein for activity is a first step to delineate those parts of the protein important for the investigated function [22-24]. The binding assays using the full-length and several truncated versions of CRE1/AHK4 (Fig. 2) confirm the hypothesis that *trans*-zeatin is bound by CRE1/AHK4 via the CHASE domain. This is relevant as cytokinin occurs also inside the plant cell and it could be, therefore, possible that cytokinin is bound also via the cytoplasmic part of the cytokinin receptors. However, our data show that this possibility is not realized in CRE1/AHK4.

### Bioinformatic analysis identifies residues that are crucial for a functional ligand binding domain

Functional important amino acid positions are often predicted by combining evolutionary information of a protein family with 3D structures. The underlying assumption is, if a position adopts a new beneficial function, it will be subject to stronger selective constraint, which will be reflected in the evolutionary rates of amino acid replacement [25]. Several methods have been described in the past that search for spatial clusters of subfamily conserved residues or that search for shifts in the evolutionary rates of protein subfamilies [26-33]. The accuracy of computer predictions has been confirmed in several cases through mutagenesis experiments [18,34,35]. In the lack of a 3D structure of the CHASE domain, we focused on identifying sites that stand out by smaller evolutionary rates in the plant subfamily or, at sites that evolve slowly in all subfamilies, by biochemical different amino acids that are conserved in the different subfamilies.

Experimental evaluation of the roles of the selected amino acids in cytokinin binding underpinned the validity of the approach. Substitutions to alanine of four of the five selected amino acids in CRE1/AHK4 showed a dramatic alteration of the cytokinin binding, in this case a strong reduction to total abolishment of the *trans*-zeatin binding (Fig. 4A), thus confirming their importance in cytokinin binding.

Interestingly, the three amino acids which show the strongest effect and also the positive control T301 are located in close vicinity in two predicted β-sheets in the center part of the CHASE domain (Fig. 1B). The only exception in this study, position K297, which, while completely conserved among plant sequences in contrast to bacterial sequences, did not show any significant reduction in cytokinin binding (Fig. 3A), is located just outside the first of these two central β-sheets. We hypothesize that these β-sheets are part of a binding pocket for cytokinins. It is understood that only the experimental determination of the structure will provide conclusive evidence. The identification of important amino acids presented here will be an important contribution to understand the functioning of the CHASE domain, once structural data become available.

### Future applications

Plant tissues typically contain a mixture of different biologically active cytokinin metabolites. The specificity of ligand recognition was investigated for *Arabidopsis *and maize cytokinin receptors and it was shown that their relative affinities for different cytokinins varies [4,16,17,36]. Analysis of the mutant receptors generated in this study has shown that the mutations do not affect the recognition of different cytokinins in a distinct way (Romanov et al., unpublished result). Thus these amino acids appear to be of general relevance. As they are conserved among all plant CHASE domains they might present the key residues for binding the hormonal core. Fine-tuning for specific cytokinins could be achieved by additional, less conserved positions. It will be interesting to see whether our bioinformatical design also enables the identification of amino acid residues which are relevant to the detection of different types of cytokinins by receptors of the same species. However, such an analysis will require a greater number of different cytokinin receptors to be studied with respect to their cytokinin binding preference.

## Conclusion

Using truncated versions of the cytokinin receptor CRE1/AHK4 in a binding assay, we have experimentally defined the CHASE domain as the ligand binding domain of this class of receptor. Based on our bioinformatical approach, combined with experimental validation, we successfully identified functionally important amino acids in this domain. The experimental confirmation of these residues highlights the significance of evolutionary proteomics in the post genomic era and demonstrates its potential for the characterization of protein functioning. In addition to the analysis of proteins with known functions, the approach can be envisioned as an aid in the enormous task of functionally annotating the vastly increasing amount of sequence information provided by the numerous genome sequencing projects.

## Methods

### Construction of the different CRE1/AHK4 variants

The truncated variants of *CRE1/AHK4 *(At2g01830.2) were generated by PCR using the respective primers (Additional file 2) and cloned into the entry vector pDONR221 of the Gateway™ cloning system (Invitrogen, Carlsbad). For the amino acid substitutions the *Hin*dIII fragment of CRE1/AHK4 containing the entire CHASE domain was cloned into pBluescript II KS (Stratagene, La Jolla). Site-directed mutagenesis was carried out with the QuikChange^® ^II Site-Directed Mutagenesis Kit (Stratagene, La Jolla) using the respective primers (Additional file 2). The mutated fragments were sequenced and recloned into a *CRE1/AHK4 *clone in the pDONR221 vector missing the *Hin*dIII fragment in the cDNA. Subsequently all clones were shuttled into the pDEST15 vector (Invitrogen, Carlsbad) and transformed into the *E. coli *strain BL21DE3pLys (Novagen, San Diego).

### Cytokinin binding assay and immunoblotting

For *in vivo *cytokinin binding the assays were performed as published [17]. Pellets of 1 ml of the respective *E. coli *culture (used in the binding assay) were resuspended in 50 μL reducing 1x Laemmli buffer [37] and heated to 95°C for 5 min before separating 25 μL of the protein extract on a 10% SDS-PAGE (Bio-Rad Laboratories, München, Germany). Proteins were transferred to PVDF membrane (Bio-Rad) using a tank transfer system (Bio-Rad) with Towbin buffer without methanol [38]. After blocking for 2 h at room temperature with Tris buffered saline (TBS) including 5% low fat dried milk powder, the membrane was incubated with anti GST primary antibody (B-14) (1:500 in blocking buffer; Santa Cruz Biotechnology, Heidelberg, Germany) 3 h at room temperature. Horseradish peroxidase-conjugated goat anti mouse secondary antibody (1:4000 in blocking buffer; Santa Cruz Biotechnology, Heidelberg, Germany) and enhanced chemiluminescence reagent (Pierce SuperSignal West Pico; Perbio Science, Bonn, Germany) were used for detection. Blots were exposed to Pierce CL-Xposure films (Perbio Science).

### Sequence analysis

Sequences containing the CHASE domain were retrieved from Genbank's non-redundant database using a Hidden Markov Model, which was built from a representative multiple sequence alignment [10] obtained from the Pfam database (HMMER package)[39]. To identify additional plant sequences, Genbank's EST database was searched with the CHASE domain of CRE1/AHK4. EST sequences were translated into proteins. Protein regions containing the CHASE domain were aligned with hmmalign (HMMER). The alignment (Additional file 1) was manually optimized to minimise gaps in loop regions and used to calculate a phylogenetic tree with CLUSTAL W [40]. Five stable subtrees with more than 90% bootstrap support were chosen for the analysis of evolutionary rates, of which one represents all plant sequences and four subtrees represent bacterial sequences. In order to identify functionally diverged amino acid sites, we estimated evolutionary rates for each position in the five alignments and compared the rates between the plant and bacterial groups. This allows the identification of positions that are under selective pressure in the plant group, while they are free of any constraint in bacterial groups. In contrast to more simple amino acid conservation scores, evolutionary rates reflect the amino acid changes considering the phylogenetic distance of the sequences. Evolutionary site rates were estimated with the maximum likelihood method implemented in the TREE-PUZZLE program v5.1 [41,42]. For the estimation of the evolutionary site rates with TREE-PUZZLE, we have chosen a heterogeneity rate model that was compared with the null model, which assumes a uniform rate among sites. The two models were compared in a log likelihood ratio test and in four out of five cases the null model was rejected (p-value < 0.01). We used the Jones Taylor amino acid substitution model and assumed an eight category discretized gamma model for the variation of substitution rates among sites. We searched for sites that are conserved in the plant group but fast evolving in bacterial groups, or that are slow evolving in all groups, but conserved in different amino acids.

## Authors' contributions

AH, KW, NN and GAR carried out the molecular studies. BP did the bioinformatic analysis. AH, BP and TS conceived the study and wrote the manuscript. KW and GAR contributed to draft the manuscript. All authors read and approved the final manuscript.

## Supplementary Material

Additional file 1 Full sequence alignment of the CHASE domain. The sequences are labelled with gi numbers and the start and end position of the subset of the sequences used for the alignment is given. In case of EST data, the complete translated sequences were used. Evolutionary site rate categories for the 5 subclasses depicted in Fig. 3 are given below the alignment. Click here for file

Additional file 2 Primers used for cloning in the different amino acid substitutions and truncation experiments. Click here for file
