# Supplementary material for: Evolutionary proteomics identifies amino acids essential for ligand-binding of the cytokinin receptor CHASE domain
Source: BMC Evol Biol. 2007 Apr 17;7:62. doi: 10.1186/1471-2148-7-62 (PMC1863423; doi:10.1186/1471-2148-7-62)
Supplement: Additional file 2 — Primers used for cloning in the different amino acid substitutions and truncation experiments. [file 1471-2148-7-62-S2.doc]

| P 105 | 5'-GCTGTCTTGACTAGCCCTGCTAGGTTGTTGGAAACTCA-3' | F_304_A-Mutagenesis |
| --- | --- | --- |
| P 106 | 5'-TGAGTTTCCAACAACCTAGCAGGGCTAGTCAAGACAGC-3' |
| P 107 | 5'-GAGCTAGAGAAACCGGAGCAGCTGTCTTGACTAGCC-3' | K_297_A-Mutagenesis |
| P 108 | 5'-GGCTAGTCAAGACAGCTGCTCCGGTTTCTCTAGCTC-3' |
| P 109 | 5'-TGTCTTGACTAGCCCTTTTGCGTTGTTGGAAACTCACCAT-3' | R_305_A-Mutagenesis |
| P 110 | 5'-ATGGTGAGTTTCCAACAACGCAAAAGGGCTAGTCAAGACA-3' |
| P 111 | 5'-CTCGGAGTTGTGTTGGCATTCCCTGTCTACAAGT-3' | T_317_A-Mutagenesis |
| P 112 | 5'-ACTTGTAGACAGGGAATGCCAACACAACTCCGAG-3' |
| P 113 | 5'-GTTTGAGCGGCAGCACAATGCGGTTATAAAGACAATGGATAG-3' | W_244_A-Mutagenesis |
| P 114 | 5'-CTATCCATTGTCTTTATAACCGCATTGTGCTGCCGCTCAAAC-3' |
| P 115 | 5'-CCGGAAAAGCTGTCTTGATTAGCCCTTTTAGGTTGTTG-3' | T_301_I-Mutagenesis |
| P 116 | 5'-CAACAACCTAAAAGGGCTAATCAAGACAGCTTTTCCGG-3' |
| P 117 | 5'-GCTACCGTGTCTCAGGAGATCAGGACACCA-3' | H_482_Q-Mutagenesis |
| P 118 | 5'-TGGTGTCCTGATCTCCTGAGACACGGTAGC-3' |
| P 78 | 5'-AGAAAGCTGGGTGTTACAGTATATAACCCACCAA-3' | AHK4 CHASE-TM |
| P 77 | 5’-AAAAAGCAGGCTCCGCTTTGATTCTGTGGATC-3’ |
| P 15 | 5’-AAAAAGCAGGCTTGGTGCGAGCAGAAGCTGC-3’ | AHK4 Cytoplasmic Domain |
| P 3 | 5'-AGAAAGCTGGGTACGACGAAGGTGAGATAG-3' |

**Supplemental Table 1:**

Primers used for cloning in the different amino acid substitutions and truncation experiments
